# Epidemiology of hip fractures: Systematic literature review of German data and an overview of the international literature

**DOI:** 10.1007/s00391-018-1382-z

**Published:** 2018-03-28

**Authors:** Kilian Rapp, Gisela Büchele, Karsten Dreinhöfer, Benjamin Bücking, Clemens Becker, Petra Benzinger

**Affiliations:** 1 Department of Clinical Gerontology, Robert-Bosch-Hospital, Auerbachstr. 110, 70376 Stuttgart, Germany; 2 Institute of Epidemiology and Medical Biometry, Ulm University, Helmholtzstr. 22, 89081 Ulm, Germany; 3 Department of Musculoskeletal Rehabilitation, Prevention and Health Service Research, Center for Sport Science and Sport Medicine (CSSB), Center for Musculoskeletal Surgery (CMSC), Charité Universitätsmedizin, Berlin, Germany; 4 Department of Orthopedics and Traumatology, Medical Park Berlin Humboldtmühle, Berlin, Germany; 5 Department of Trauma, Hand and Reconstructive Surgery, University of Giessen and Marburg GmbH, Baldingerstraße, 35043 Marburg, Germany

**Keywords:** Risk factors, Mortality, Intellectual disability, Institutionalization, Prevention, Risikofaktoren, Mortalität, Geistige Behinderung, Institutionalisierung, Prävention

## Abstract

**Background:**

Hip fractures are regarded as a worldwide epidemic and a major public health concern. Changing risk factors, local differences and temporal trends contribute to the particular epidemiology of hip fractures. This overview gives a comprehensive insight into the epidemiology of hip fractures and reviews where German data have contributed to the literature.

**Methods:**

The review of the epidemiology of hip fractures in Germany is based on a systematic literature search in PubMed. Information about the global epidemiology of hip fractures was provided by a selective literature review focusing on specific aspects of the epidemiology of hip fractures.

**Results:**

Hip fracture rates vary more than 100-fold between different countries. In most high-income countries, a rise in age-standardized hip fracture rates was observed until the 1980s and 1990s and a decrease thereafter. Such a decrease has not been observed for Germany so far. Many factors, diseases and drugs have been found to be associated with hip fractures and there is some evidence that fracture risk in later life is already programmed during fetal life and early childhood. Of the hip fracture burden 50% occur in people with disability and in need of care. In nursing homes approximately 4 fractures can be expected in 100 women per year. In people with intellectual or developmental disabilities comparable risks of hip fracture occur 10–40 years earlier than in the general population. Incidence of disability, institutionalization and death are frequent consequences of hip fractures.

**Conclusion:**

The epidemiology of hip fractures is characterized by a high burden of disease, local differences, temporal trends, well-defined high-risk populations and many established risk factors.

****Electronic supplementary material**:**

The online version of this article (10.1007/s00391-018-1382-z) contains supplementary material, which is available to authorized users.

## Zusammenfassung

**Hintergrund:**

Hüftfrakturen werden weltweit als eines der großen Gesundheitsprobleme angesehen. Diese Übersicht vermittelt einen umfassenden Einblick in die Hüftfrakturepidemiologie und zeigt, wo deutsche Daten neue Erkenntnisse hinzugefügt haben.

**Methode:**

Das Review zur deutschen Hüftfrakturepidemiologie basiert auf einer systematischen Literatursuche in PubMed. Informationen zu spezifischen Teilaspekten der globalen Hüftfrakturepidemiologie beruhen auf einer selektiven Literatursuche.

**Ergebnisse:**

Hüftfrakturraten unterscheiden sich um mehr als das 100-fache zwischen verschiedenen Ländern. In den meisten westlichen Industriestaaten wurde ein Anstieg der altersstandardisierten Raten bis in die 80er- und 90er-Jahre und ein Rückgang in den Folgejahren beobachtet. In Deutschland wurde bisher kein Rückgang der Hüftfrakturrate beobachtet. Hüftfrakturen treten in 50 % der Fälle bei Personen mit Pflegebedürftigkeit auf. In Pflegeheimen muss jährlich mit 4 Hüftfrakturen pro 100 Bewohnern gerechnet werden. Bei Personen mit geistiger Behinderung treten Hüftfrakturen 10 bis 40 Jahre früher auf als in der Allgemeinbevölkerung. Pflegebedürftigkeit, Institutionalisierung und Tod sind häufige Folgen von Hüftfrakturen.

**Schlussfolgerung:**

Die Hüftfrakturepidemiologie ist durch eine hohe Krankheitslast, geographische Unterschiede, zeitliche Veränderungen, Hochrisikopopulationen und eine Reihe etablierter Risikofaktoren charakterisiert.

## Introduction

The life expectancy of the world population is increasing and more and more people are reaching high ages. This results in a shift of the health burden towards diseases appearing predominantly in higher ages. Hip fractures occur in old and very old people and the absolute number of fractures is therefore strongly affected by the observed demographic change. In the meantime, hip fractures are regarded as a worldwide epidemic and a major public health concern in many countries [1]. Globally, during the year 2000, there were an estimated 1.6 million hip fractures [2] accounting for about 20% of all fractures in people aged 50 years and older. Since nearly all people with a hip fracture are hospitalized for surgical treatment, most of the fractures are captured by routine data. This is an excellent basis for epidemiological analyses and resulted in a large body of literature. There are considerable geographic differences in the incidence of hip fractures which cannot be explained by different age structures of the studied populations. In addition, heterogenous secular trends in the incidence of hip fractures have been observed during the last decades. These local differences and temporal trends contribute to the particular epidemiology of hip fractures.

This overview gives a comprehensive insight in the complete spectrum of global hip fracture epidemiology. Furthermore, the overview points out which epidemiological data from Germany are available, how they differ from other studies and in which fields they add new aspects to the literature of hip fracture epidemiology.

## Methods

For the review of hip fracture epidemiology derived from German data a systematic literature search in PubMed was performed (for search strategy see Electronic supplementary material). The literature search identified 145 manuscripts and 8 further manuscripts were found by manually searching the reference lists. After evaluation of title, abstract or full text, 131 publications did not meet the inclusion and exclusion criteria. Despite one inclusion criterion, which was a publication date in 2000 or later, we added 5 articles published before 2000 due to their relevance. The German studies are presented in Supplementary Table 1.

To provide an overview of the international hip fracture epidemiology, an additional selective literature review was performed to identify publications with specific aspects of hip fracture epidemiology.

## Results

### Age and sex

The incidence of hip fractures increases exponentially with age. A decrease in bone mass and an increase in falls result in the strong association between age and the risk of hip fractures [3]. In Western countries approximately three out of four hip fractures occur in women. This huge difference in the absolute number of fractures is partly explained by the higher life expectancy of women. The age-standardized difference between women and men is lower with a relation of about 2:1 in most countries of the world [3, 4]. In Germany, the age-standardized relationship between women and men is somewhat lower (1.72:1) and even nearly identical if residents of German nursing homes are compared (ratio 1.26:1; data derived from Rapp et al.; [5]). Male residents have clearly higher fall rates than female residents [6], which may contribute to their similar risk of hip fractures in long-term care.

### Geographic differences

Hip fracture rates differ considerably between different countries and regions of the world. The rates vary more than 200-fold in women and more than 140-fold in men [7]. The countries with the highest incidence are northern Europe (Norway, Sweden, Iceland, Ireland) followed by Central Europe (Denmark, Belgium, Germany, Switzerland, Austria) and eastern Europe (Czech Republic, Slovakia, Hungary) and the Middle East (Oman, Iran). Other high-risk countries are Argentina and Taiwan [8]. The reasons for the huge geographic differences in hip fracture incidence are not well understood. Secular trends in hip fracture incidence which are described below in more detail and migration studies suggest environmental rather than genetic reasons [8]. Various country indicators, such as socioeconomic status, development and urbanization are positively correlated with hip fracture risk [7]; however, there is no clear evidence about an association between socioeconomic factors and fracture risk within countries [9]. Urban areas have 20–60% higher incidences of hip fractures than rural areas [10]. Hard surfaces due to soil sealing, lower physical activity and lower serum levels of vitamin D due to less sun exposure may contribute to the higher risks in urban areas and in countries with higher socioeconomic prosperity. Germany is a high-risk country for hip fractures with an incidence of approximately 130 fractures/100,000 citizens per year (standardized to the German population; [11]). Considerable differences in hip fracture incidence have been reported for the different federal states [12]. They do not follow an apparent pattern and the underlying reasons remain unclear. In addition, no consistent pattern was observed between area level socioeconomic conditions and hip fracture risk [13].

### Secular trends

Age-specific hip fracture rates have changed considerably over time in most analyzed countries [14]. The majority of available studies are based on data from Western countries. For most of the regions there was a steep rise in age-standardized rates until the 1980s and 1990s and a decrease thereafter. The trends have been more pronounced in women than in men [15]. For populous regions like South America or many parts of Asia continuously rising hip fracture rates are reported. These trends are highly relevant since they strongly influence the national hip fracture burden. A decrease in age-specific hip fracture rates could counteract the predicted increase due to demographic changes in high income countries [16].

The reasons for the increase and decrease of hip fracture rates within short time periods are speculative. The rapid increase in hip fracture risk is paralleled with the process of urbanization which may act through lower physical activity, more hard surfaces, less sun exposure or other life style factors [14]. An increasing survival of frail people who have low bone quality and a high risk of falls may also contribute to the trend. Factors proposed for the declining trend are the ‘compression of morbidity’ with higher physical activity and lower fall rates, increasing rates of obesity and the introduction of anti-resorptive drugs. The hip fracture risk today may be also influenced by conditions during pregnancy and childhood. Similar to the Barker hypothesis which suggests an association between intrauterine undernourishment and coronary heart disease [17], there is some evidence that fracture risk in later life is already programmed during fetal life and early childhood [18]. Low weight and size at birth and poor childhood growth have been observed to be related to low peak bone mass and high fracture risk later in life [19]. Therefore, a cohort effect may additionally contribute to recent changes of hip fracture risk reflecting changing conditions in nourishment and life style from many years ago.

In the former Eastern Germany an increase in the age-standardized hip fracture rate of 3% per year was observed from 1974 to 1989 [20]. Data for the former Western Germany are not available for this time period. After reunification age-standardized hip fracture rates were approximately 10% and 20% lower in both women and men of the former Eastern Germany, respectively [21]. Between 1995 and 2010 there was no significant trend in the total German population; however, different trends were observed in different subgroups with decreasing rates in women of former Western Germany and increasing rates in men of former Western and Eastern Germany [22]. The converging incidence rates in the former Eastern and Western Germany may be explained by the converging life styles in both parts of Germany [21].

### Change of risk over short time periods

**Fig. 1:**
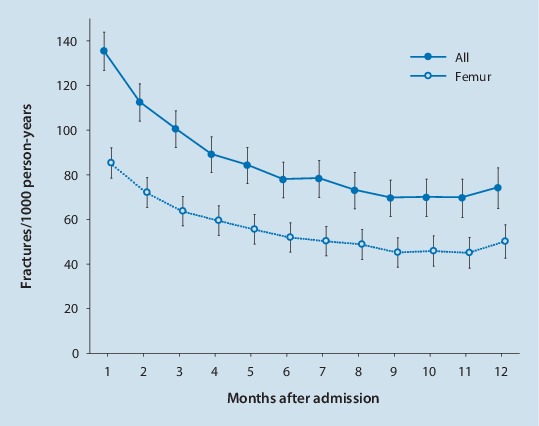
Incidence rate of fractures as a function of time since admission to a nursing home for all fractures combined and femoral fractures. (Data from Rapp et al. [26])

The individual fracture risk can change within short time periods. It is well known that hip fracture risk is increased immediately after a preceding fracture [23, 24]. In two German studies it was observed that the initial time period after admission to a nursing home is a high-risk situation for hip and other fragility fractures [25, 26]. The fracture risk was highest during the first weeks after admission and declined thereafter (Fig. 1). Potential causes of the observed pattern may be the new environment which is a challenge to many of the new and often cognitively impaired residents. Another German study found that patients hospitalized due to any reason had an increased risk for hip fractures during the first weeks after discharge from hospital to their homes [27]. A morbidity-related weakness with a deterioration of gait and balance, and a persisting (sub-acute) delirium may be further reasons for a transient increased risk of falls and fractures.

### Seasonal patterns

Several studies reported higher incidence rates of hip fractures during winter months [28]. This seasonal pattern was also observed in countries without snow and ice [29]. Low outside temperature, precipitation and wind are associated with increased fracture rates [28].

### Risk factors

Many factors, diseases and drugs have been found to be associated with hip fractures. More than 90% of hip fractures are caused by a fall [30] and two thirds of patients with a hip fracture have osteoporosis [31]. Therefore, established risk factors usually work by influencing fall risk and bone quality or both. Age and female sex are strongly associated with fracture risk [3]. The association with a parental history of hip fractures shows that a hereditary component contributes to the hip fracture risk [32]. Further established factors are prior fractures [33], falls, low muscle strength, underweight and smoking [1]. Some diseases are strongly associated with fracture risk. Examples are Cushing’s disease, hyperthyroidism and diabetes mellitus type 1 [34–36]. Other diseases have been also found to be associated with fractures, such as depression or epilepsy [37, 38]. Drugs, such as glucocorticoids and aromatase inhibitors may also influence bone mass and bone quality or increase fall risk like benzodiazepines [39], anti-depressants or anti-psychotics [40]. For more comprehensive lists of risk factors we refer to the literature [41].

Three German studies [42–44] support earlier findings [45–47] of an increased risk of hip fractures in people with Parkinson’s disease, after stroke, or in patients with dementia.

### High-risk populations

**Fig. 2:**
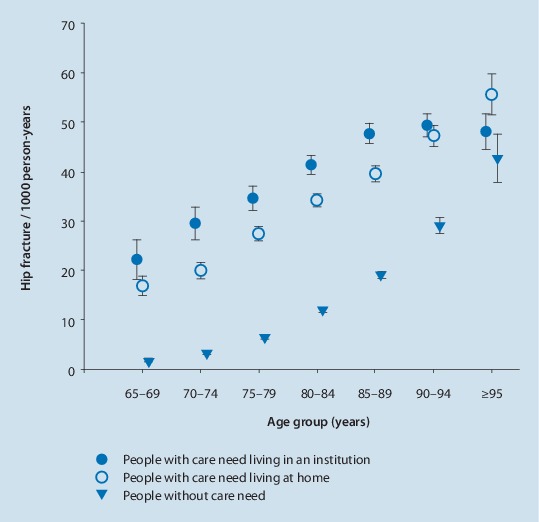
Femoral fracture rates in women stratified by setting (community-dwelling people without care needs, community-dwelling people with care needs and residents of nursing homes) and age. (Data from Rapp et al. [5])

Fracture risk is particularly high in people with disabilities. German data show that 50% of the hip fracture burden occurs in people with disabilities and need for care living at home or in an institution. In younger age groups (65–80 years) the risk of hip fracture is up to 10 times higher in people with care needs than in people without care needs ([5]; Fig. 2).

Long-term care institutions are the setting with the highest fracture risk. One of the most valid datasets about the incidence of hip fractures occurring in long-term care institutions derives from a German dataset with nearly 70,000 residents [25]. Approximately 4 and 3 hip fractures can be expected in women and men per 100 resident places per year, respectively [5, 25]; however, within this very frail population the degree of disability and fracture risk is inversely associated [25]. Former studies have shown that people with intellectual or developmental disabilities (DD) often have a low bone mass density and an increased fall risk [48]. Some of the discussed reasons are gait problems, seizures, medication or endocrine disorders [48, 49]. Data from Germany demonstrated that comparable risks of hip fracture occur about 10–15 years earlier in females and even 20–40 years earlier in males with DD than in the general population [50].

### Consequences of hip fractures

#### Mortality

Hip fractures occur predominantly in frail older people who have a high baseline mortality risk. Many studies have demonstrated that hip fractures additionally increase the risk of death. Excess mortality is consistently higher in men than in women [51]. It is highest in the days and weeks following the fracture and remains elevated for months [51, 52]. It is estimated that 20–30% of deaths are causally related to the fracture event [53]. In residents of German nursing homes excess mortality during the first 6 months is even higher with 57.8% in men and 32.9% in women [25].

#### Disability

Hip fractures have a high impact on older people’s abilities, function and quality of life. Only 40–60% of hip fracture patients recover their prefracture level of mobility [54]. Between 20% and 60% of patients who were independent in self-care activities, such as washing and dressing before the fracture require assistance to do these tasks after 1 year [55]. Most patients who recover their prefracture function and walking ability do so within the first 6 months after discharge from hospital [54, 56]. The degree of recovery is even lower in institutionalized patients.

#### Institutionalization

**Fig. 3:**
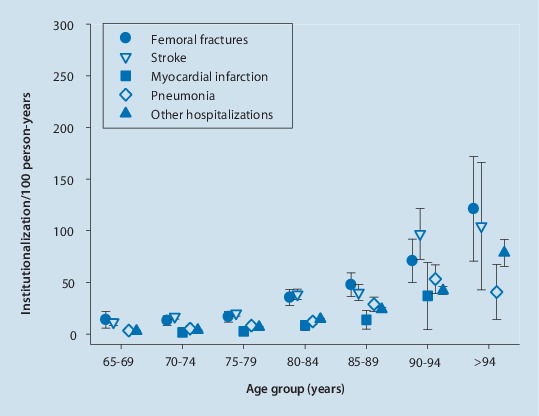
Rates of institutionalization within 6 months after discharge from hospital in men with femoral fracture, stroke, myocardial infarction, pneumonia and other reasons for hospitalization in different age categories. (Data from Rapp et al. [57])

Hip fractures may compromise an independent life and make it often impossible to live at home any longer. In high income countries, 10–20% of hip fracture patients are institutionalized following a hip fracture [54]. A German study demonstrated institutionalization rates of 15% in women and 11.8% in men within 6 months after hospital discharge [57]. The risk of institutionalization increases from 3.6% in women aged 65–69 years to 34.8% in women aged 95 years and older. In men the risk of institutionalization after hip fracture is even comparable in size with that after stroke (Fig. 3).

#### Secondary fractures

A prior hip fracture increases the risk of a subsequent fragility fracture by the factor 2– 2.5 [58, 59]. The increase of the relative risk is similar in men and women. The risk for a second hip fracture is particularly pronounced during the first months after the first fracture [60].

### Prevention of hip fractures

The etiology of hip fractures is complex and the underlying factors are only partly amenable to prevention. Lifelong moderate to vigorous physical activity seems to reduce the risk of hip fractures by about 40% [61]. Osteoporosis can be treated by specific medication which reduces the risk of hip fractures by about 40% [62]; however, a considerable percentage of people with a new hip fracture do not meet the criteria of treatment prior to the fracture [31]. Single fall prevention studies were not sufficiently powered for the analysis of fracture incidence but a meta-analysis of fall prevention exercise interventions found a reduction of osteoporotic fractures by 61% [63]; however, a model calculation which used German baseline conditions showed that unrealistically high medical treatment rates or fall prevention participation rates are needed to achieve substantial effects on the burden of hip fractures at present and in the future [31]. Therefore, coordinated interventions are needed which address different measures and strategies of fracture prevention on a population level [64].

## Caption Electronic Supplementary Material

Hip fracture epidemiology based on German data
