# Supplementary material for: Epidemiology of hip fractures: Systematic literature review of German data and an overview of the international literature
Source: Z Gerontol Geriatr. 2018 Mar 28;52(1):10–6. doi: 10.1007/s00391-018-1382-z (PMC6353815; doi:10.1007/s00391-018-1382-z)
Supplement: Supplementary file 1 — Hip fracture epidemiology based on German data [file 391_2018_1382_MOESM1_ESM.docx]

SUPPLEMENTS

**Supplement ‘Search strategy’**

On 10 February 2017 a literature search in PubMed was performed with the following search strategy: (((((hip fractures[Title/Abstract]) OR femoral fractures[Title/Abstract]) OR hip fracture[Title/Abstract]) OR femoral fracture[Title/Abstract])) AND ((Germany[Title/Abstract]) OR German[Title/Abstract]).

Articles were included if the following criteria were met:

- data of German origin

- primary data (registers, routine data from health care providers or health care insurances, data from the federal statistics office)

- information on hip fracture epidemiology

- data covering persons aged 65 years and older

- published after 1st January 2000

Articles were excluded if any of the following criteria were met:

- very small and non-representative samples

- data on surgical treatment, in-hospital treatment and post-acute treatment

- reported data which based on results originally published by another study

Supplement Table 1: Hip fracture epidemiology based on German data published a) between 2000 and 2016 and b) before 2000

| **Publication** | **Observation period** | **Setting** | **Participants** | **Data source** | **Outcome of interest** |
| --- | --- | --- | --- | --- | --- |
| **a) published between 2000 and 2016** | |  |  |  |  |
| Büchele et al., 2017 (50) | 2008 - 2010 | general population Baden-Württemberg | persons with developmental disabilities;  age group: 70 years and younger;  N = 17,880 | hospital discharge data held by health insurance (AOK Bavaria) | hip fracture incidence |
| Bluhmki et al., 2017 (33) | January 1st 2004 to June 30th 2009 | general population  Bavaria | patients admitted to hospital with hip fracture;  age groups: 65 years and older ;  median age 74.0 years (women); 76.4 years (men);  N = 872,390 | hospital discharge data held by health insurance (AOK Bavaria) | incidence and mortality with / without previous index fracture |
| Abendroth & Möhrke, 2016 (S1) | 2000 - 2013 | general population | total German population;  age groups: 50 years and older;  N = 29 million to 34 million | national hospital discharge diagnosis register, census, official statistics | hip fracture incidence over time |
| Bohlken et al., 2015 (46) | 2010 - 2013 inclusion, follow up to April 30th 2015 | general population | primary care practices;  patients with a new diagnosis of dementia and free of a fracture history, matched controls;  age groups: 65 to 90 years,  mean age 81.3 years;  N = 53,156/53,156 | electronic practice records | fracture-free survival |
| Rapp et al., 2015 (57) | baseline January 1st 2005, follow up to June 30th 2009 | community-dwelling persons  Bavaria | patients admitted to hospital with hip fracture;  age groups: 65 years and older at baseline, median age 77.2 years;  N = 414,049 | hospital discharge data held by health insurance (AOK Bavaria) | institutionalisation after hip fracture |
| Benzinger et al., 2015 (44) | January 1st 2003 to June 30th 2009 | general population Bavaria | persons admitted to hospital with primary diagnosis of stroke;  age groups: 65 years and older, median age 78.9 years;  N = 78,461 | hospital discharge data held by health insurance (AOK Bavaria) | hip fracture incidence |
| Defèr et al., 2014 (12) | 2000-2011 | general population | age groups: 50 years and older | structured hospital discharge data | hip fracture incidence, stratification by federal state |
| Benzinger et al., 2014 (42) | 2003 - 2009 | general population  Bavaria | patients with Parkinson's disease;  age groups: 65 years and older, median age 77.9 years;  N = 23,469 | hospital discharge data held by health insurance (AOK Bavaria) | hip fracture incidence |
| Requena et al., 2014 (S2) | 2006 - 2008 | general population Bavaria | patients of general practitioners' or specialists' practices;  age groups: 50 years and older;  N = 3,885,264/3,938, 210/3,988,146 | German Bavarian association of statutory health physicians database | hip fracture incidence |
| Müller-Mai et al., 2015 (S3) | 2007 - 2008 | general population | persons admitted to hospital with proximal femoral fracture;  age groups: all  mean age 81.5 years;  N = 7,905 | hospital discharge data held by health insurance (Knappschaft Bahn See) | mortality, changes in living situation and care need after hip fracture |
| Icks et al., 2013 (22) | 1995 - 2010 | general population | total German population;  age groups: all;  N = 81.5 to 81.7 million | national hospital discharge diagnosis register, census, official statistics | hip fracture incidence stratified by East / West Germany |
| Rapp et al., 2012 (27) | January 1st 2004 to June 30th 2009 | general population  Bavaria | persons discharged from hospital;  age groups: 65 years and older;  median age 76.3 (women) / 73.5 (men) ;  N = 692,347 | hospital discharge data held by health insurance (AOK Bavaria) | hip fracture incidence |
| Rapp et al., 2012 (5) | January 1st 2004 to June 30th 2009 | general population  Bavaria | age groups: 65 years and older  median age 73.6 (women) / 71.5 (men) ;  N = 932,197 | hospital discharge data held by health insurance (AOK Bavaria) | hip fracture incidence, stratified according to need for nursing care |
| A. Defèr, et al., 2011 (S4) | 2000 - 2009 | general population | total German population;  N = 82.3 million to 81.9 million | national hospital discharge diagnosis register, census, official statistics | hip fracture incidence |
| A. Icks et al., 2009 (13) | 1994 - 2010 | general population | total German population;  age groups: all;  N = 81.8 million | national hospital discharge diagnosis register, census, official statistics | associations between hip fracture incidence and socioeconomic conditions |
| Rapp et al., 2009 (26) | 2001 - 2006 | nursing home residents,  Bavaria | persons newly admitted to nursing homes;  age groups: 65 years and older, median age 84.3 (women) / 80.8 (men) years;  N = 93,424 | hospital discharge data held by health insurance (AOK Bavaria) | hip fracture rates as a function of time after admission to nursing home |
| Rapp et al., 2008 (25) | 2000 - 2005 | nursing home residents,  Baden-Württemberg | persons newly admitted to nursing homes;  age groups: 65 years and older, median age 85.2 (women) / 81.5 (men) years;  N = 69,692 | hospital discharge data held by health insurance (AOK Baden-Württemberg) | hip fracture incidence in nursing homes;  association between functional impairment and hip fracture incidence;  hip fracture rates as a function of time after admission to nursing home;  excess mortality caused by hip fracture |
| Icks et al., 2008 (11) | 1995 - 2004 | general population | total German population;  age groups: all;  N = 81.8 million | national hospital discharge diagnosis register, census, official statistics | hip fracture incidence over time |
| Endres et al., 2006 (S5) | January 1st 2002 to September 30th 2003 | general population | patients admitted to acute or postacute hospitals (N = 423);  age groups: all, mean age 77.5 years;  N = 12,520 (31.3 % acute hospitals, 68.9 % post-acute hospitals) | hospital data plus post-discharge interviews | clinical parameters, mortality up to one and a half years after discharge, changes in mobility and living situation |
| Hoffmann & Glaeske, 2006 (S6) | January 1st 2004 to December 31st 2004 | general population | age groups: all;  N = 636 | hospital discharge data held by health insurance (Gmünder Ersatzkasse) | hip fracture incidence |
| Smektala et al., 2005 (S7) | 1999 | general population,  region of Westfalen-Lippe | patients admitted to hospital with femoral fractures;  age groups: all;  N = 1,353 | structured hospital quality data, routine data of health insurance and long-term care insurance | institutionalisation, mortality |
| Wildner & Clark, 2001 (21) | 1996 | general population | total German population, 10% random sample | structured hospital discharge data | hip fracture incidence stratified by federal states |
| **b) published before 2000** | | | | | |
| Wildner M, Casper W, Bergmann KE. 1999 (20) | | | | | |
| Becker C, et al., 1999 (S8) | | | | | |
| Wildner M et al. 1998 (S9) | | | | | |
| Wildner M, Casper W, Bergmann KE. 1997 (S10) | | | | | |
| Cöster A, Haberkamp M, Allolio B. 1994 (S11) | | | | | |

**References Supplement Table 1**

5. Rapp K, Becker C, Cameron ID, Klenk J, Kleiner A, Bleibler F, et al. Femoral fracture rates in people with and without disability. Age Ageing. 2012 Sep;41(5):653–8.

6. Rapp K, Becker C, Cameron ID, König H-H, Büchele G. Epidemiology of falls in residential aged care: analysis of more than 70,000 falls from residents of bavarian nursing homes. J Am Med Dir Assoc. 2012;13(2):187–e1.

11. Icks A, Haastert B, Wildner M, Becker C, Meyer G. Trend of hip fracture incidence in Germany 1995–2004: a population-based study. Osteoporos Int. 2008;19(8):1139–45.

12. Defèr A, Schober H-C, Möhrke W, Abendroth K, Hofbauer LC, Task Group for Treatment Research at German Society of Osteology, Federal Association of German Osteologists (Bundesverband der Osteologen Deutschland e.V.), et al. Are there still east-to-west differences in the incidence of hip fractures in Germany? Arch Osteoporos. 2014;9:195.

13. Icks A, Haastert B, Wildner M, Becker C, Rapp K, Dragano N, et al. Hip fractures and area level socioeconomic conditions: a population-based study. BMC Public Health. 2009 Apr 27;9:114.

20. Wildner M, Casper W, Bergmann KE. A secular trend in hip fracture incidence in East Germany. Osteoporos Int J Establ Result Coop Eur Found Osteoporos Natl Osteoporos Found USA. 1999;9(2):144–50.

21. Wildner M, Clark DE. Hip fracture incidence in east and west germany: reassessement ten years after unification. Osteoporos Int J Establ Result Coop Eur Found Osteoporos Natl Osteoporos Found USA. 2001;12(2):136–9.

22. Icks AIA, Arend WAW, Becker CBC, Rapp KRK, Haastert BHB. Incidence of hip fractures in Germany, 1995–2010. Arch Osteoporos. 2013;8(1-2):1–7.

25. Rapp K, Becker C, Lamb SE, Icks A, Klenk J. Hip fractures in institutionalized elderly people: incidence rates and excess mortality. J Bone Miner Res Off J Am Soc Bone Miner Res. 2008 Nov;23(11):1825–31.

26. Rapp K, Lamb SE, Klenk J, Kleiner A, Heinrich S, König H-H, et al. Fractures after nursing home admission: incidence and potential consequences. Osteoporos Int. 2009 Oct;20(10):1775–83.

27. Rapp K, Cameron ID, Becker C, Kleiner A, Eckardt M, König H-H, et al. Femoral fracture rates after discharge from hospital to the community. J Bone Miner Res Off J Am Soc Bone Miner Res. 2012 Nov 5;

31. Benzinger P, Becker C, Todd C, Bleibler F, Rothenbacher D, König H-H, et al. The impact of preventive measures on the burden of femoral fractures – a modelling approach to estimating the impact of fall prevention exercises and oral bisphosphonate treatment for the years 2014 and 2025. BMC Geriatr. 2016 Apr 1;16:75.

33. Bluhmki T, Peter RS, Rapp K, König H-H, Becker C, Lindlbauer I, et al. Understanding Mortality of Femoral Fractures Following Low-Impact Trauma in Persons With and Without Care Need. J Am Med Dir Assoc. 2017 Mar 1;18(3):221–6.

42. Benzinger P, Rapp K, Maetzler W, König H-H, Jaensch A, Klenk J, et al. Risk for Femoral Fractures in Parkinson’s Disease Patients with and without Severe Functional Impairment. Toft M, editor. PLoS ONE. 2014 May 22;9(5):e97073.

44. Benzinger P, Rapp K, König HH, Bleibler F, Globas C, Beyersmann J, et al. Risk of osteoporotic fractures following stroke in older persons. Osteoporos Int. 2015 Apr;26(4):1341-9.

46. Bohlken J, Jacob L, Schaum P, Rapp MA, Kostev K. Hip fracture risk in patients with dementia in German primary care practices. Dementia (London). 2017 Oct;16(7):853-864.

50. Büchele G, Becker C, Cameron ID, Auer R, Rothenbacher D, König HH, et al. Fracture risk in people with developmental disabilities: results of a large claims data analysis. Osteoporos Int. 2017 Jan;28(1):369-375.

57. Rapp K, Rothenbacher D, Magaziner J, Becker C, Benzinger P, König H-H, et al. Risk of Nursing Home Admission After Femoral Fracture Compared With Stroke, Myocardial Infarction, and Pneumonia. J Am Med Dir Assoc. 2015 Aug;16(8):715.e7–715.e12.

S1. Abendroth K, Möhrke W. Anzahl und Inzidenz der Hüftfrakturen in Deutschland von 2000 bis 2013. Ist aus epidemiologischen Daten dieser Periode eine Hüftfrakturprävention durch die Osteoporosetherapie ableitbar? Osteologie. 2016;25(1):12–20.

S2. Requena G, Abbing-Karahagopian V, Huerta C, De Bruin ML, Alvarez Y, Miret M, et al. Incidence Rates and Trends of Hip/Femur Fractures in Five European Countries: Comparison Using E-Healthcare Records Databases. Calcif Tissue Int. 2014 Jun;94(6):580–9.

S3. Müller-Mai CM, Schulze Raestrup US, Kostuj T, Dahlhoff G, Günster C, Smektala R. Einjahresverläufe nach proximalen Femurfrakturen: Poststationäre Analyse von Letalität und Pflegestufen durch Kassendaten. Unfallchirurg. 2015 Sep;118(9):780–94.

S4. Defèr A, Möhrke W, Abendroth K. Zehnjahrestrend in der Inzidenz der Hüftfrakturen in Deutschland von 2000 bis 2009. In der Gesamtpopulation und der Altersgruppe mit erhöhtem Osteoporoserisiko. Osteologie. 2011;20(4):1019–29.

S5. Endres HG, Dasch B, Lungenhausen M, Maier C, Smektala R, Trampisch HJ, et al. Patients with femoral or distal forearm fracture in Germany: a prospective observational study on health care situation and outcome. BMC Public Health. 2006 Apr 4;6:87.

S6. Hoffmann F, Glaeske G. Inzidenz proximaler Femurfrakturen in Deutschland. Gesundheitswesen. 2006 Mar 31;68(03):161–4.

S7. Smektala R, Ohmann C, Paech S, Neuhaus E, Rieger M, Schwabe W, et al. [On the prognosis of hip fractures. Assessment of mortality after hip fractures by analyzing overlapping segments of longitudinal data]. Unfallchirurg. 2005 Nov;108(11):927–8, 930–7.

S8. Becker C, Fleischer S, Hack A, Hinderer J, Horn A, Scheible S, et al. [Disabilities and handicaps due to hip fractures in the elderly]. Z Gerontol Geriatr. 1999 Oct;32(5):312–7.

S9. Wildner M, Markuzzi A, Casper W, Bergmann K. [Disparities in hospital mortality after proximal femoral fractures in East Germany 1989]. Soz Praventivmed. 1998;43(2):80–9.

S10. Wildner M, Casper W, Bergmann KE. Estimating the incidence of hip fractures in East Germany from hospital discharge statistics. J Epidemiol Community Health. 1997 Oct;51(5):576–7.

S11. Cöster A, Haberkamp M, Allolio B. [Incidence of femoral neck fractures in the German Federal Republic in comparison to other countries]. Soz Praventivmed. 1994;39(5):287–92.
